# Inhibition of IL-17 and IL-23 in Human Keratinocytes by the A_3_ Adenosine Receptor Agonist Piclidenoson

**DOI:** 10.1155/2018/2310970

**Published:** 2018-05-15

**Authors:** Shira Cohen, Faina Barer, Inbal Itzhak, Michael H. Silverman, Pnina Fishman

**Affiliations:** Can-Fite BioPharma Ltd., 10 Bareket St., Kiryat Matalon P.O. Box 7537, 49170 Petah-Tikva, Israel

## Abstract

Interleukin-17 and interleukin-23 play major roles in the inflammatory process in psoriasis. The Gi protein-associated A_3_ adenosine receptor (A_3_AR) is known to be overexpressed in inflammatory cells and in peripheral blood mononuclear cells (PBMCs) of patients with autoimmune inflammatory conditions. Piclidenoson, a selective agonist at the A_3_AR, induces robust anti-inflammatory effect in psoriasis patients. In this study, we aimed to explore A_3_AR expression levels in psoriasis patients and its role in mediating the anti-inflammatory effect of piclidenoson in human keratinocyte cells. A_3_AR expression levels were evaluated in skin tissue and PBMCs derived from psoriasis patients and healthy subjects. Proliferation assay and the expression of signaling proteins were used to evaluate piclidenoson effect on human keratinocytes (HaCat). High A_3_AR expression levels were found in a skin biopsy and in PBMCs from psoriasis patients in comparison to healthy subjects. Piclidenoson inhibited the proliferation of HaCat cells through deregulation of the NF-*κ*B signaling pathway, leading to a decrease in interleukin-17 and interleukin-23 expression levels. This effect was counteracted by the specific antagonist MRS 1523. A_3_AR overexpression in skin and PBMCs of psoriasis patients may be used as a target to inhibit pathological cell proliferation and the production of interleukin-17 and interleukin-23.

## 1. Introduction

Psoriasis, affecting 2-3% of the worldwide population, is an inflammatory, chronic skin disease considered to be an immune-mediated one. It is characterized by excessive proliferation and abnormal differentiation, accompanied by apoptosis of keratinocytes (KCs) causing erythematous, scaly patches, or plaques on the skin [1–3]. In the psoriatic skin area, expression of proinflammatory cytokines and chemokines is upregulated, attracting immune cells leading to the proliferation of local and invading cells [4]. The proinflammatory cytokine interleukin-23 (IL-23) is one of the major cytokines involved in the pathogenesis of psoriasis [5] by mediating Th17 cells, which release interleukin-17 (IL-17) into the inflamed environment. IL-23 is highly expressed in psoriatic skin and is secreted among other cells, also from KCs [6]. Its blockade by injection of specific human anti-IL-23 antibody results in neutralizing IL-23 and prevention of disease progression in psoriasis patients [7]. High levels of IL-17 have been detected in psoriatic skin lesions [8, 9], strengthening the assumption that inhibition of both cytokines will result in improvement of disease pathology.

A_3_ adenosine receptor (A_3_AR) is a Gi protein-coupled cell surface receptor, known to mediate anti-inflammatory effects upon activation with selective agonists. The A_3_AR was found to be highly expressed in inflammatory tissues [10, 11] and in the peripheral blood mononuclear cells (PBMCs) [12–14] of patients with Crohn's disease, psoriasis, and rheumatoid arthritis (RA). The mechanism responsible for receptor overexpression suggests the involvement of the transcription factors nuclear factor kappa B (NF-*κ*B) and cyclic adenosine monophosphate (cAMP) response element-binding protein (CREB), both present in the A_3_AR gene promotor and are known to be upregulated in psoriasis [12]. It was found that the inflammatory cytokine tumor necrosis factor *α* (TNF-*α*) regulates A_3_AR via an autocrine pathway while an increase in this cytokine expression results in a subsequent increase in NF-*κ*B and receptor upregulation [13].

Piclidenoson is an A_3_AR highly specific agonist, inducing a robust anti-inflammatory effect demonstrated in preclinical pharmacology studies in rheumatoid arthritis, osteoarthritis, uveitis, and colitis [14–19]. This anti-inflammatory effect is mediated via downregulation of the NF-*κ*B signaling pathway resulting in TNF-*α* inhibition [14, 16].

The efficacy of piclidenoson in plaque psoriasis has been demonstrated in phase II clinical studies [15, 20].

In the present study, we have further investigated the expression of the A_3_AR in cells from psoriasis patients and explored the utilization of A_3_AR as a therapeutic target.

## 2. Materials and Methods

### 2.1. Reagents

The A_3_AR agonist 1-deoxy-1-[6-[[(iodophenyl)methyl]amino]-9H-purine-9-yl]-N-methyl-(-D-ribofuranuronamide), piclidenoson (also known as IB-MECA), was synthesized by Can-Fite BioPharma. A stock solution of 10 mM was prepared in dimethyl sulfoxide (DMSO) and further diluted in phosphate-buffered saline (PBS). The A_3_AR antagonist MRS 1523 was purchased from Sigma (St. Louis, MO, USA) and diluted in the same manner as for piclidenoson. Dulbecco's Modified Eagle's medium (DMEM) 4.5 g/L glucose, fetal bovine serum (FBS), and antibiotics for cell cultures were obtained from Beit HaEmek, Haifa, Israel. Polyclonal antibodies against human A_3_AR, phosphoinositide 3-kinase (PI3K), NF-*κ*B, TNF-*α*, IL-17, IL-23, and *β*-actin were purchased from Santa Cruz Biotechnology Inc., Ca, USA. Monoclonal antibody against human p-AKT was purchased from Cell Signaling Technology, MA, USA.

### 2.2. Patients and Healthy Subject Population

To analyze A_3_AR expression levels, blood samples were collected from 48 psoriasis patients with moderate to severe disease that participated in a phase 2 study as part of the protocol (NCT00428974) [5]. Blood samples from 50 healthy subjects were collected upon ethical committee approval. All donors signed an informed consent prior to blood withdrawal. To separate PBMCs, the blood (16 ml) was collected in BD Vacutainer® CPT tubes (BD, Franklin Lakes, NJ, USA) and centrifuged according to the manufacturer's instructions and washed with PBS.

### 2.3. RT-PCR Analysis of Formalin-Fixed Paraffin-Embedded Skin Biopsy

RNA expression levels in psoriasis formalin-fixed paraffin-embedded skin biopsy were detected as previously described [21]. In brief, the formalin-fixed, paraffin-embedded skin biopsy tissue was purchased from a tissue bank. To differentiate between the psoriasis tissue and normal skin regions, slides were stained with H&E and observed by a pathologist. Later on, nonstained sequential slides were marked for psoriasis and normal area based on the stained slides.

Tissue sections (20 *μ*m) were mounted on slides that were stained by hematoxylin and eosin (H&E) and were observed by a pathologist. Tissue sections on slides were deparaffinized in xylene and rehydrated by washing in serial dilutions of ethanol. Slides were used immediately or stored at −80°C until used. After rehydration, 20 *μ*l of solution A [1.25X PCR buffer (200 mM Tris–HCl and 500 mM KCl), 6.25 mM MgCl2, 5 units RNasin (Promega, Madison, WI, USA), 2 mM DTT, and 1 unit RQ1 RNase-free DNase (Promega)] was applied directly to the marked area. The psoriatic skin lesion and the adjacent tissue were completely scraped off the slide using a pipette tip and were collected to a different microcentrifuge tube. The samples were treated with proteinase K at a final concentration of 0.1 mg/ml. The samples were incubated at 37°C for 1 hour to allow for DNA digestion. Cells lysate were heated to 95°C for 15 min to inactivate DNase and proteinase K. After centrifugation at 14,000 rpm for 5 min, 17 *μ*l of the supernatant was transferred to a separate tube, and 4 *μ*l of reverse transcription mixture [5 mM deoxynucleoside triphosphate, 2.5 *μ*M random hexamer, 5 units RNasin, 100 units SuperScript One-Step RT-PCR with Platinum Taq (Invitrogen), and the primers for A_3_AR 5′-ACGGTGAGGTACCACAGCTTGTG and 3′ ATACCGCGGGATGGCAGACC] was added. The reverse transcription reaction was performed at 45°C for 45 min followed by heating to 99°C for 5 min. Next, 50 cycles of 94°C for 30 s, 59°C for 45 s, and 73°C for 45 s were performed. Products were electrophoresed on 2% agarose gels, stained with ethidium bromide (Et-Br), and visualized with UV illumination. The specificity of the RT-PCR reaction was confirmed by size determination on agarose gels in comparison with a positive control, from RNA extracted using standard techniques, and by sequencing the RT-PCR product and comparing the sequences to that of the known sequences (ADORA3- L77729, L77730). The absorbance of the bands (Et-Br) was quantified using an image analysis system. To quantitate A_3_AR mRNA expression, the absorbance value was normalized against the cell number in each psoriatic lesion or healthy skin tissue.

### 2.4. Cell Culture

HaCat cells derived from human KCs (AddexBio, San Diego, CA, USA) were grown in DMEM 4.5 g/L glucose medium containing penicillin, streptomycin, 2 mM L-glutamine, and 10% FBS. The cells were maintained in T-75 flasks at 37°C in a 5% CO^2^ incubator and transferred to a freshly prepared medium twice weekly. For all studies, serum-starved cells were used. FBS was omitted from the cultures for 18 hours, and the experiment was carried out on monolayers of cells in DMEM 4.5 g/L glucose medium supplemented with 1% FBS in a 37°C and 5% CO^2^ incubator.

### 2.5. [^3^H]-Thymidine Incorporation Assay

[^3^H]-Thymidine incorporation assay was used to evaluate cell growth. HaCat cells (2500 cells/well) were incubated with piclidenoson 10 nM and MRS 1523 (50 nM) in 96-well plate for 48 hours. For the last 24 hours of incubation, each well was pulsed with 1 mCi [^3^H]-thymidine. Cells were harvested, and the [^3^H]-thymidine uptake was determined in an LKB liquid scintillation counter (LKB, Piscataway, NJ, USA). These experiments were repeated at least 4 times.

### 2.6. Western Blot Analysis

To detect expression levels of A_3_AR, PI3K, p-AKT, NF-*κ*B, TNF-*α*, IL-17, IL-23, and *β*-actin, the protein extract from piclidenoson-treated or -untreated HaCat cells was utilized. Cells were incubated in the presence and absence of piclidenoson (10 nM) for 48 hours at 37°C. At the end of the incubation period, cells were then rinsed with ice-cold PBS and transferred to ice-cold lysis buffer (TNN buffer, 50 mM Tris buffer pH = 7.5, 150 mM NaCl, NP 40 0.5% for 20 min). Cell debris was removed by centrifugation for 10 min, at 7500  ×g. The supernatant was utilized for Western blot analysis. Protein concentrations were determined using the NanoDrop (Thermo Scientific). Equal amounts of the sample (50 *μ*g) were separated by SDS-PAGE, using 4–12% polyacrylamide gels. The resolved proteins were then electroblotted onto nitrocellulose membranes (Pall Corporation, Florida, USA). Membranes were blocked with 5% bovine serum albumin and incubated with the desired primary antibody (dilution 1 : 1000) for 24 hours at 4°C. Blots were then washed and incubated with a secondary antibody for 1 hour at room temperature. Bands were recorded using BCIP/NBT color development kit (Promega, Madison, WI, USA). Densitometry of protein expression was normalized against *β*-actin and expressed as % of control.

### 2.7. A_3_AR mRNA Expression Level Analysis

For quantification of A_3_AR levels from psoriasis patients, blood samples were withdrawn to BD Vacutainer CPT™ tube with sodium citrate. Cells were separated according to the manufactory protocol. Total RNA was extracted using the RNeasy mini kit (QIAGEN) with QIAshredder Spin Columns (QIAGEN). RNA (500 ng) was reverse transcribed by high-capacity cDNA reverse transcription kit (AB Applied Biosystems) according to the manufactory protocol.

Real-time PCR was performed using rotor gene 3000 RT-PCR detection system (Corbett Research, Australia) according to manufacturer's instruction. Briefly, The PCR primer used was 5′- GGCCAATGTTACCTACATCAC -3′ [forward] and 5′-CAGGGCTAGAGAGACAATGAA-3′ [reversed] for ADORA3. Processes were set as follows: initial denaturation at 95°C for 10 min, 30 cycles of amplification including a denaturation step at 95°C for 15 sec, an annealing step at 50°C, and an extension step at 72°C for 15 sec. Direct detection of PCR products monitored by measuring the fluorescence produced by the result of TaqMan probe hydrolysis after every cycle.

### 2.8. Statistical Analysis

The results were evaluated using the Student's *t*-test and analysis of variance (ANOVA) test with statistical significance set at *p* < 0.05. Comparison between the mean values of different experiments was carried out. All data are reported as mean ± SD.

## 3. Results

### 3.1. A_3_AR Is Overexpressed in Skin Tissue and PBMCs of Psoriasis Patients

**Figure 1:**
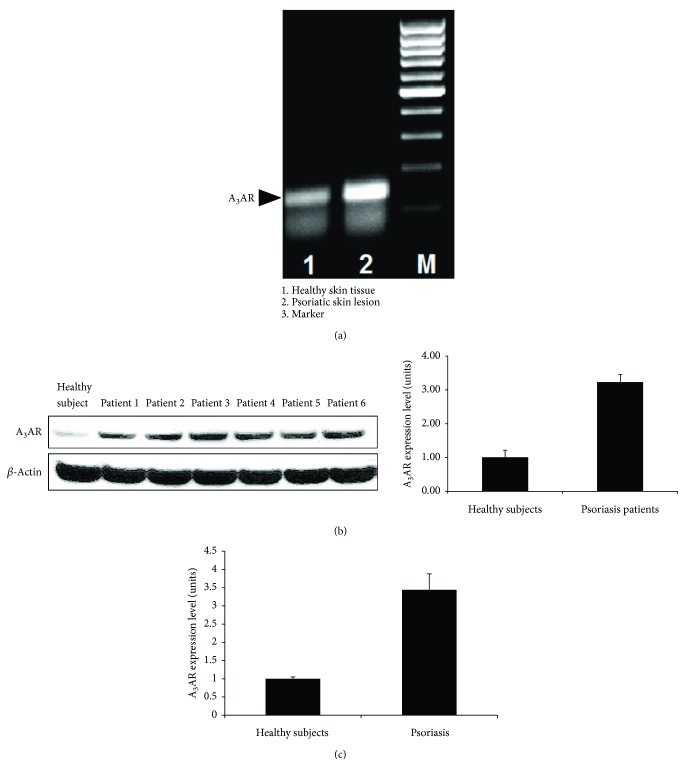
A_3_AR overexpression in a skin lesion and PBMCs from psoriasis patients. (a) A_3_AR expression level was evaluated in formalin-fixed paraffin-embedded representative skin lesion from psoriasis patient. A_3_AR mRNA in the psoriatic skin lesion was compared to adjacent healthy skin tissue using RT-PCR. A_3_AR was highly expressed in the skin lesion compared to the healthy tissue. (b) A_3_AR protein expression level was analyzed using WB analysis in PBMCs from psoriasis patients (*n* = 48) compared with healthy subjects (*n* = 50). A significant (*p* = 0.0001) A_3_AR upregulation is noted. (c) A_3_AR mRNA expression level was tested in PBMCs from psoriasis patients compared to healthy subjects using real-time PCR.

High expression levels of A_3_AR were found in a representative psoriatic skin lesion compared to the normal adjacent tissue within the skin biopsy (Figure 1(a)). High A_3_AR protein expression level was found in 97% of 48 PBMCs samples of psoriasis patients in comparison to 50 samples from healthy subjects.  Figure 1(b) is a representative blot from 6 patients versus healthy subject showing an increase of ~3.4 fold in A_3_AR expression levels compared to the naive (*p* < 0.0001). Overexpression of A_3_AR mRNA was also detected in the patients' PBMCs showing an increase of ~3.5 fold compared to that of the healthy subjects (Figure 1(c)).

### 3.2. Piclidenoson Inhibits the Proliferation of HaCat Cells

**Figure 2:**
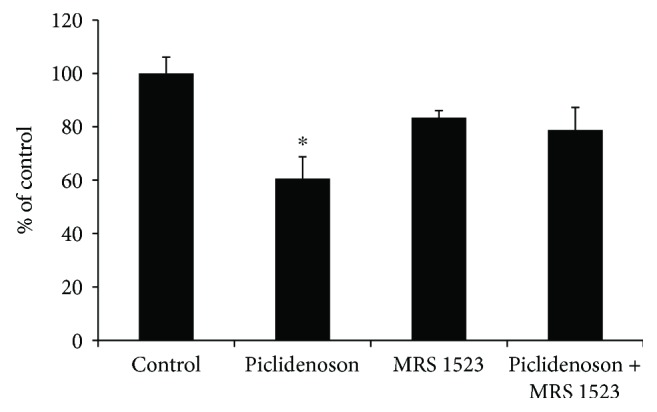
Piclidenoson inhibits the proliferation of HaCat cells. Proliferation assay was performed on HaCat cells that were incubated in the presence of piclidenoson (10 nM) and A_3_AR antagonist MRS 1523 (50 nM) for 48 hours (^∗^*p* < 0.05).

Piclidenoson inhibits the proliferation of HaCat cells by 40% ±8.1. Introduction of the antagonist MRS 1523 to the culture system counteracted piclidenoson inhibitory effect indicating that the response was A_3_AR mediated (*p* < 0.01) (Figure 2).

### 3.3. Piclidenoson De-Regulates Cell Growth Regulatory Proteins

**Figure 3:**
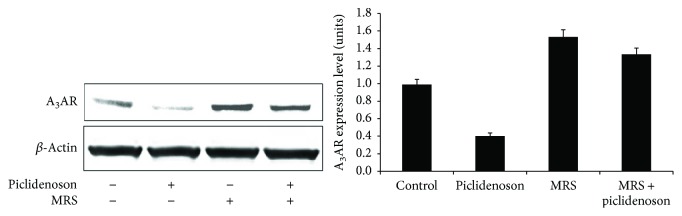
Piclidenoson downregulates A_3_AR protein expression level. HaCat cells were incubated in the presence and absence of piclidenoson and MRS 1523 for 48 hours. Downregulation of A_3_AR was demonstrated upon piclidenoson treatment and reversed by the introduction of MRS 1523 to the culture.

**Figure 4:**
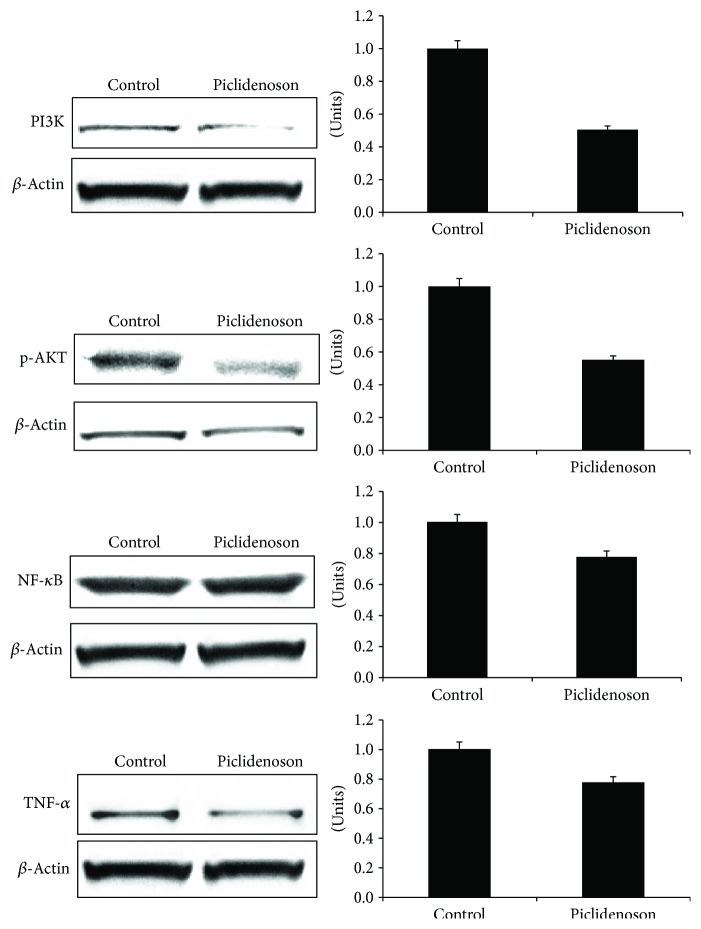
Piclidenoson downregulates the NF-*κ*B signaling pathway. HaCat cells were incubated in the presence and absence of piclidenoson (10 nM) for 48 hours. The expression level of PI3K, p-AKT, NF-*κ*B, and TNF-*α* was tested by Western blot analysis.

Piclidenoson induced a decrease in A_3_AR expression level, demonstrating a response to the agonist. The specificity of this response was shown by the antagonist MRS 1523 which counteracted this effect (Figure 3). Downstream to receptor activation, a decrease in the expression level of PI3K, p-AKT, NF-*κ*B, and TNF-*α* took place (Figure 4). Based on our former experience, the total AKT has not been modulated upon treatment with piclidenoson in inflammatory cells [14].

### 3.4. Piclidenoson Inhibits IL-17 and IL-23

**Figure 5:**
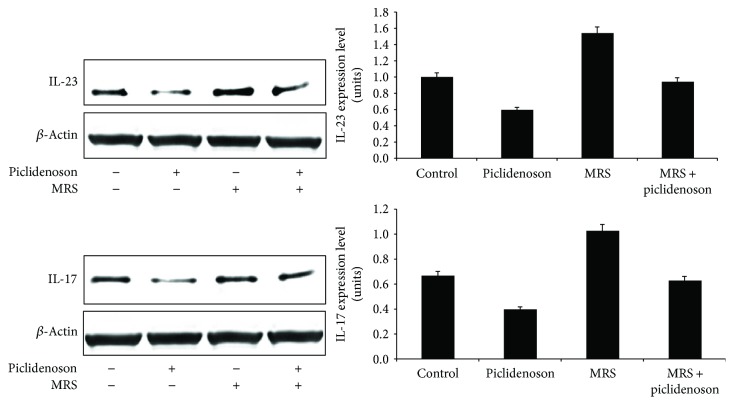
Piclidenoson downregulates IL-17 and IL-23. HaCat cells were incubated in the presence and absence of piclidenoson (10 nM) and MRS 1523 (50 nM) for 48 hours. The expression level of the inflammatory cytokines IL-17 and IL-23 was tested by Western blot analysis.

Piclidenoson inhibits IL-17 and IL-23 expression levels whereas MRS 1523 reversed the inhibitory effect (Figure 5).

## 4. Discussion

In this study, we further demonstrate the overexpression of A_3_AR in a skin lesion and in the PBMCs of psoriasis patients with moderate to severe disease. Targeting the receptor with the highly specific agonist piclidenoson, A_3_AR induced the inhibition of HaCat cell proliferation, a spontaneously immortalized human epithelial line that maintains full epidermal differentiation capacity and has been widely used as an *in vitro* model for the study of psoriasis [22, 23]. Moreover, piclidenoson induced a decrease in the expression level of PI3K, p-AKT, NF-*κ*B, TNF-*α*, IL-17, and IL-23, known to act as potent inflammatory mediators in psoriasis.

It has been reported earlier that A_3_AR is overexpressed in the PBMCs of patients with autoimmune inflammatory conditions such as crohn's disease, rheumatoid arthritis, and psoriasis, compared to healthy subjects [12]. Interestingly, A_3_AR overexpression was found to be directly correlated to NF-*κ*B and CREB, two transcription factors present in the A_3_AR gene promoter. NF-*κ*B is one of the most important mediators in the pathogenesis of psoriasis. It is a transcription factor which belongs to the Rel1 family and is involved in the regulation and function of different proinflammatory genes. It has been found that many of the triggering factors for psoriasis evolve via the activation of NF-*κ*B which subsequently translocate to the epidermis and basal cells, resulting in epidermal hyperplasia and inflammation, thereby affecting KCs proliferation and differentiation [22]. The involvement of a large surface skin area in psoriasis patients intensifies the role of factors such as NF-*κ*B and TNF-*α*, leading to amplification of the inflammatory process, which may also attribute to A_3_AR overexpression.

A_3_AR was found to be overexpressed in the psoriasis skin lesion biopsy in comparison to the normal adjacent tissue. Also, mRNA A_3_AR expression levels were ~3.5 fold higher in the PBMCs of the psoriasis subjects versus healthy subjects. These findings corroborate with former data showing that high A_3_AR expression in the inflammatory target organ is mirrored in the patients' PBMCs [12].

A support for the utilization of the A_3_AR as a therapeutic target came from the data of the present study showing that piclidenoson inhibited the proliferation of HaCat cells.

In the keratinocyte cultures, shortly after A_3_AR activation with piclidenoson, receptor protein expression was downregulated, a response known to initiate downstream molecular events leading to cell proliferation inhibition [10]. This downregulation was mostly reversed by introducing the antagonist MRS 1523 to the culture, indicating that piclidenoson effect is A_3_AR mediated. Receptor downregulation is a general mechanism typical of Gi protein receptors. This family of receptors responds to ligand activation by receptor internalization (to the cytosol), degradation, resynthesis, and recycling to the cell surface [24]. During these events, receptor desensitization/resensitization takes place and different signaling pathways are initiated [25]. We suggest that the downregulation of receptor expression in this study represents the rapid response of the HaCat cells to agonist stimulation and the initiation of downstream signaling events.

Subsequent to the deregulation of NF-*κ*B and related proteins, a decrease in the expression levels of TNF-*α*, IL-17, and IL-23 took place, further strengthening the utilization of piclidenoson as a drug candidate to combat psoriasis. By adding MRS 1523 to the culture system, the inhibition of IL-17 and IL-23 by piclidenoson was abolished suggesting that A_3_AR acts as a modulator for these cytokines. Activation of NF-*κ*B and TNF-*α* induces IL-23 production [26, 27], therefore suggesting that the inhibitory effect of piclidenoson on NF-*κ*B and TNF-*α* may result in deregulation of IL-23 and IL-17. Monoclonal antibodies against TNF-*α*, IL-17, and IL-23 were found to mediate a strong anti-inflammatory effect that has been already implemented for the treatment of psoriasis patients and proved to be highly efficacious [25, 27–31].

## 5. Conclusion

Piclidenoson, a selective A_3_AR agonist, inhibits KCs proliferation via the deregulation of the NF-*κ*B signal transduction pathway resulting in a decrease in the expression levels of IL-17 and IL-23. Piclidenoson has previously been tested in 2 phase II clinical studies showing an anti-inflammatory effect and good safety profile [15, 32]. The current data further support the development of this drug candidate for the treatment of psoriasis.

## Abbreviations

Kcs: Keratinocytes
IL-23: Interleukin-23
IL-17: Interleukin-17
A_3_AR: A_3_ adenosine receptor
PBMCs: Peripheral blood mononuclear cells
RA: Rheumatoid arthritis
NF-*κ*B: Nuclear factor kappa B
CREB: cAMP response element-binding protein
TNF-*α*: Tumor necrosis *α*
PI3K: Phosphoinositide 3-kinase.
